# Dancing to Another Tune—Adhesive Moonlighting Proteins in Bacteria

**DOI:** 10.3390/biology3010178

**Published:** 2014-03-10

**Authors:** Veera Kainulainen, Timo K. Korhonen

**Affiliations:** 1 Department of Veterinary Biosciences, University of Helsinki, P.O. Box 66, FI-00014 Helsinki, Finland; E-Mail: veera.kainulainen@helsinki.fi; 2 General Microbiology, Department of Biosciences, University of Helsinki, P.O. Box 56, FI-00014 Helsinki, Finland

**Keywords:** moonlighting proteins, bacterial adhesins, surface anchoring, secretion, decaying cells, enolase, glyceraldehyde-3-phosphate dehydrogenase, GroEL, *Lactobacillus*

## Abstract

Biological moonlighting refers to proteins which express more than one function. Moonlighting proteins occur in pathogenic and commensal as well as in Gram-positive and Gram-negative bacteria. The canonical functions of moonlighting proteins are in essential cellular processes, *i.e.*, glycolysis, protein synthesis, chaperone activity, and nucleic acid stability, and their moonlighting functions include binding to host epithelial and phagocytic cells, subepithelia, cytoskeleton as well as to mucins and circulating proteins of the immune and hemostatic systems. Sequences of the moonlighting proteins do not contain known motifs for surface export or anchoring, and it has remained open whether bacterial moonlighting proteins are actively secreted to the cell wall or whether they are released from traumatized cells and then rebind onto the bacteria. In lactobacilli, ionic interactions with lipoteichoic acids and with cell division sites are important for surface localization of the proteins. Moonlighting proteins represent an abundant class of bacterial adhesins that are part of bacterial interactions with the environment and in responses to environmental changes. Multifunctionality in bacterial surface proteins appears common: the canonical adhesion proteins fimbriae express also nonadhesive functions, whereas the mobility organelles flagella as well as surface proteases express adhesive functions.

## 1. Introduction—Concept of Moonlighting Proteins

Moonlighting proteins include an expanding class of adhesion proteins in both prokaryotic and eukaryotic organisms. They are multifunctional and go against the general one gene-one protein-one function hypothesis. Moonlighting proteins possess two or more separate functions that cannot be ascribed to gene fusions, splice variants, or protein fragments expressing different functions after proteolysis (see the excellent review by Copley [1]). Moonlighting offers an individual protein a mechanism to increase network complexity and to participate in apparently unrelated cellular functions [2]. Moonlighting proteins were first described in eukaryotic cell biology 25 years ago, with the identification of the cytokine neuroleukin as phosphoglucose isomerase (PGI) and of a lens structural protein as enolase, *i.e.*, as cytoplasmic enzymes of glucose metabolism [3,4]. Extracellular production of a glycolytic enzyme, invertase, in several yeast species was reported already in 1960s [5]. In bacteria, the first identified moonlighting enzyme was glyceraldehyde 3-phosphate dehydrogenase (GAPDH)—also a glycolytic enzyme—that was found in the cytoplasm as well as on the cell surface of group A streptococci. This bacterium, *Streptococcus pyogenes*, inhabits human throat and skin and is responsible for a variety of non-invasive and invasive infections, and GAPDH was found to exhibit *in vitro* multiple adhesive functions with a potential to increase bacterial virulence [6]. Since then, several moonlighting proteins have been identified or suggested in eukaryotic and prokaryotic organisms. In bacteria alone, the current number of protein species with an identified adhesive moonlighting function exceeds 20, and the on-going proteomic studies will undoubtedly reveal many more. As discussed in this review, practically all enzymes in the Embden-Mayerhof glycolytic pathway of bacteria have been assigned with an adhesive moonlighting function. In addition to adhesiveness, bacterial moonlighting proteins may influence other aspects of bacterial physiology. The glycolytic enzymes also moonlight by forming complexes with themselves as well as with proteins that function in RNA processing and such complexes are thought to potentiate metabolic channelling and glycolytic fluxes within the cell [7].

Moonlighting proteins often perform their canonical and moonlighting functions in separate cell compartments, e.g., the bacterial cytoplasm and the cell surface, and therefore such a dual cellular localization of a protein is suggestive of moonlighting. This is a limited criterium but has remained common in identification of a bacterial moonlighting protein, especially in proteomic studies. Detailed analyses of genes encoding moonlighting proteins, the proteins and their domains as well as functions in bacteria are few, whereas *in vitro* studies demonstrating unexpected adherence or binding partners for a moonlighting protein are more numerous. Here, we will address those bacterial moonlighting proteins that have been characterized for an adhesive function as well as for a cellular location. Our main emphasis is on their interactions with mammalian host components as well as on their anchorage on the bacterial surface. For general reviews on moonlighting proteins, the reader is referred to recent reviews [1,2,8,9].

In bacteria, the first moonlighting proteins were identified in pathogenic Gram-positive bacteria and shown to have pathogenesis-related functions, which stressed their importance as putative virulence factors (reviewed by Henderson and Martin [10]). However, recent research has also demonstrated moonlighting proteins in Gram-negative and in non-pathogenic or commensal bacteria as well. This indicates that moonlighting is common in bacteria. Most of the currently known moonlighting proteins and functions, however, come from Gram-positive bacteria (see Table 1), which may reflect their less complex cell wall structure and easier protein translocation. Bacteria differ in immobilization of moonlighting proteins on their surface which can seriously affect the function of the protein in different organisms (discussed below). A single bacterial strain can harbor several different moonlighting proteins, and a moonlighting protein species can express several distinct moonlighting functions. On the other hand, the same moonlighting function can be provided by different proteins [1]. Canonical bacterial adhesion proteins, such as the fimbriae, exhibit functions that are not directly involved in adherence to host tissues or cells, and, on the other hand, bacterial flagella as well as surface-associated proteases possess adhesive properties. These proteins are not generally included in discussion of bacterial moonlighting, probably because they are multimeric and perform their functions in the same cellular compartment, *i.e.*, the bacterial surface. Their diverse functions however illustrate that multitasking is common in bacterial surface proteins, and we will end our review on a short description of fimbriae, flagella, and surface proteases.

## 2. Bacterial Moonlighting Proteins Come in Many Forms

Table 1 lists characterized bacterial moonlighting proteins with adhesive interactions, their organisms, and their primary and moonlighting functions. In general, moonlighting proteins in a bacterial species have been studied individually, independently from those in other bacterial species, which complicates obtaining a comprehensive and mechanistic view on bacterial moonlighting. The currently known bacterial moonlighting proteins have their canonical function in essential cellular processes, such as glycolysis, chaperone activity, protein synthesis, and nucleic acid stability (Table 1). This means that silencing of a moonlighting-associated gene most likely has broad phenotypic effects that can be hard to interpret in terms of either the canonical or the moonlighting function. The adhesive moonlighting functions that have been recognized thus far are diverse and include binding to secreted mucins, epithelial cells, lymphocytes and monocytes, extracellular matrices, circulating effector molecules, and other microbes (Table 1). The physiological significance and biological consequences of these interactions are known only in few cases. The structure of most bacterial moonlighting proteins, such as enolase and GAPDH, are highly conserved across the kingdoms and do not markedly differ in prokaryotes or eukaryotes, which is understandable in view of their essential metabolic functions and the fact that they moonlight in both organisms. Our limited knowledge of structure-function relationships in bacterial moonlighting proteins has limited the prediction of possible moonlighting functions in proteins; however, bioinformatic approaches for such predictions have been initiated [11,12].

**Table 1:** Bacterial moonlighting proteins binding to host components.

Moonlighting protein	Moonlighting function	Bacterial species	Reference
**Metabolic enzymes**			
**GAPDH**			
	Binding to plasmin(ogen)	*B. anthracis*, *E. coli*, *La. crispatus*, *La. plantarum*, *Li. monocytogens*, *S. aureus*, *St. agalactiae*, *St. anginosus*, *St. epidermis*, *St. equisimilis*, *St. oralis*, *St. pneumoniae*, *St. pyogenes*, *St. suis*	[6,13,14,15,16,17,18,19,20,21,22,23,24]
	Binding to urokinase receptor on human pharyngeal cells	*St. pyogenes*	[25]
	Binding to lysozyme	*St. pyogenes*	[6]
	Binding to actin	*St. pyogenes*, *St. agalactiae*	[6,22]
	Binding to myosin	*St. pyogenes*	[6]
	Binding to albumin	*St. suis*	[26]
	Binding to fibrinogen	*St. agalactiae*	[22]
	Binding to fibronectin	*E. coli*, *L. plantarum*, *St. pyogenes*	[6,14,16,27]
	Binding to other bacterial species	Group B *Streptococcus*, *La. crispatus*	[28,29]
	Binding to intestinal epithelial cells, competitive exclusion and displacement of *Clostridium sporogenes* and *Enterococcus faecalis*	*La. plantarum*	[30]
	Coadhesin of *Porfyromonas gingivalis* in periodontal tissue	*St. oralis*	[31]
	Binding to intestinal epithelial cells	*E. coli*, *La. plantarum*	[14,16]
	Binding to colonic, porcine or vaginal mucin	*La. plantarum*, *M. genitalium*	[16,32,33]
	Binding to A and B blood antigens	*La. plantarum*	[34]
	Binding to porcine tracheal rings	*St. suis*	[35]
	EGF-receptor	*M. avium*, *M. tuberculosis*	[36]
	Binding to C5a complement protein	*St. pyogenes*	[37]
**Enolase**			
	Binding to plasmin(ogen)	*B. anthracis*, *Bi. animalis* subspecies *lactis*, *Bi. bifidum*, *Bi. breve*, *Bi. longum*, *Bo. burgdorferi*, *La. crispatus*, *La. johnsonii*, *La. plantarum*, *Li. monocytogenes*, *M. pneumoniae*, *S. aureus*, *St. anginosus*, *St. mutans*, *St. oralis*, *S. pneumoniae*, *S. pyogenes*	[16,17,19,21,38,39,40,41,42,43,44,45,46,47,48]
	Binding to fibronectin	*La. plantarum*	[16,49]
	Binding to laminin	*La. crispatus*, *La. johnsonii*, *S. aureus*	[48,50]
	Binding to collagen	*La. crispatus*, *S. aureus*	[48]
	Binding to albumin	*St. pyogenes*	[51]
	Binding to salivary mucin	*St. gordonii*, *St. mutans*	[42,52]
	Binding to intestinal epithelial cells	*La. plantarum*, *St. suis*	[16,53]
	Binding to C4b-binding proteins	*St. pneumoniae*	[54]
	Binding to other bacterial species	*La. crispatus*	[28]
**Aldolase**			
	Binding to flamingo cadherin	*St. pneumoniae*	[55]
**Glucose-6-phosphate isomerase (GPI)**			
	Binding to collagen	*La. crispatus*	[28]
	Binding to other bacterial species	*La. crispatus*	[28]
**Phosphofructokinase**			
	Binding to plasmin(ogen)	*St. oralis*	[19]
**Phosphoglycerate kinase**			
	Binding to plasmin(ogen)	*St. anginosus*, *St. oralis*, Group B streptococci	[19,56]
	Binding to actin	Group B streptococci	[56,57]
**Phosphoglycerate mutase**			
	Binding to plasmin(ogen)	*Bi. animalis* subspecies *lactis*, *St. anginosus*, *St. oralis*	[19,58]
**Triosephosphate isomerase**			
	Binding to plasmin(ogen)	*St. anginosus*, *St. oralis*	[19]
	Binding to intestinal epithelial cells, competitive exclusion and displacement of *Clostridium sporogenes* and *Enterococcus faecalis*	*La. plantarum*	[30]
**Glutamine synthetase**			
	Binding to plasmin(ogen)	*Bi. animalis* subspecies *lactis*, *La. crispatus*, *M. tuberculosis*	[28,58,59]
	Binding to fibronectin	*M. tuberculosis*	[59]
	Binding to collagen I and laminin	*La. crispatus*	[28]
	Binding to other bacterial species	*La. crispatus*	[28]
**Ribonucleotide reductase**			
	Binding to plasmin(ogen)	*S. aureus*	[44]
**Inosine 5′-monophosphate dehydrogenase (IMPDH)**			
	Binding to plasmin(ogen)	*S. aureus*	[44]
**Alcohol acetaldehyde dehydrogenase**			
	Binding to Caco-2 cells	*Li. monocytogenes*	[60]
	Binding to eukaryotic Hsp60	*Li. monocytogenes*	[61]
**Malate synthase**			
	Binding to laminin and fibronectin	*M. tuberculosis*	[62]
**SarA; oligopeptide-binding protein**			
	Binding to salivary mucin	*St. gordonii*	[52]
**Pyruvate dehydrogenase **			
	Binding to fibronectin	*La. plantarum*	[47]
	Binding to plasmin(ogen)	*M. pneumoniae*	[46]
	Binding to fibronectin	*M. pneumoniae*	[63]
**Puryvate kinase**			
	Binding to salivary mucin	*St. gordonii*	[52]
**Bile salt hydrolase**			
	Binding to plasmin(ogen)	*Bi. animalis* subspecies *lactis*	[58]
**Molecular chaperones**			
**DnaK**			
	Binding to plasmin(ogen)	*Bi. animalis* subspecies *lactis*, *M. tuberculosis*, *Li. monocytogenes*	[21,58,59,64]
	Stimulation of dendritic cell maturation by binding CCR5	*M. tuberculosis*	[65]
	Competition with HIVfor CCR5 binding	*M. tuberculosis*	[65,66]
	Mediation of LAB adherence to yeast cells	*L. lactis*	[67]
**GroEL**			
	Binding to intestinal HT-29 cells and mucus; stimulation of IL-8 secretion in human macrophages and HT-29 cells; aggregation of *H. pylori* cells	*La. johnsonii*	[68]
**Translational elongational factors**			
**EF-Tu**			
	Binding to plasmin(ogen)	*Li. monocytogenes*, *Ps. aeruginosa*	[21,69]
	Binding to plasma Factor H and Factor H-related protein 1 (FHR-1)	*Ps. aeruginosa*	[69]
	Binding to intestinal epithelial cells and HT-MTX-derived mucus	*La. johnsonii*	[70]
	Binding to salivary mucin	*St. gordonii*	[52]
	Binding to intestinal epithelial cells, competitive exclusion and displacement of *Clostridium sporogenes* and *Enterococcus faecalis*	*La. plantarum*	[30]
	Binding to fibronectin	*M. pneumoniae*	[63]
**EF-G**			
	Binding to salivary mucin	*St. gordonii*	[52]
**Other proteins**			
**Ag85 complex of *M. tuberculosis***			
	Binding of plasmi(ogen)	*M. tuberculosis*	[59]
	Binding to fibronectin	*M. tuberculosis*	[59]
**DNA-directed RNA polymerase beta´subunit**			
	Binding to salivary mucin	*St. gordonii*	[52]
**Endopeptidase O**			
	Binding of plasmin(ogen)	*St. pneumoniae*	[71]
	Binding to fibronectin	*St. pneumoniae*	[71]
	Binding and invasion to epithelial and endothelial cells	*St. pneumoniae*	[71]
**SecA**			
	Binding to salivary mucin	*St. gordonii*	[52]
**Superoxide dismutase **			
	Binding to epithelial cell ldolase, GAPDH and cyclophilin A	*M. avidum*	[72]

## 3. How Can the Separate Functions Be Arranged in a Moonlighting Protein?

Commonly, the canonical and the moonlighting functions are provided by separate parts of the protein. Alternatively, they can be partially overlapping or provided by alternative conformations of the protein. The latter may involve post-translational modifications, binding of ligands, a pH change, expression in a different cell type or a cellular location, immobilization on a surface, oligomerization, or other perturbation [1,51,73]. Binding of GAPDH of *Lactobacillus plantarum* to human colonic mucins is inhibited by exogenous NAD, which was inferred to indicate that the catalytic region of the enzyme is involved in the adhesion [32]. The well characterized enolase of the severe pathogen *Streptococcus pneumoniae* has multiple adhesive properties which include binding of the human circulating protease precursor, plasminogen [39]. Plasminogen harbors lysine-binding kringle domains, and the interaction with pneumococcal enolase apparently takes place via two pairs of lysines in enolase, whose mutations influence plasminogen binding [47,74,75]. The two lysines in the sequence ^248^FYNKDDHKY are exposed on the surface of the enolase octamer [76], and the mutations affect the enzymatic activity only partially or not at all, which suggests that the enzymatic and the adhesive functions do not involve overlapping regions. Amino acid sequences similar to this pneumococcal plasminogen-binding motif are present in other bacterial enolases as well [43,75]. For example, the genome of *Lactobacillus johnsonii* contains three copies of the enolase gene which are 50%–72% identical to each other and encode sequences homologous to the plasminogen-binding motif of pneumococcal enolase [77]. Denaturation of the octameric form of pneumococcal enolase exposes C-terminal lysine residues that also bind plasminogen, which gives an example of a bacterial moonlighting function depending on a variation of protein conformation. However, terminal lysine residues are not present in most of the enolase proteins of lactobacilli that, however, show binding to plasminogen [48,78]. Moonlighting enzymes are released from the surface of lactic acid bacteria under stress conditions (see below), and hence small conformational changes may be relevant in their biology. Kornblatt and coworkers [51] studied *in vitro* the interaction between plasminogen and Group A streptococcal enolase by a wealth of biophysical and biochemical analyses and concluded that the interaction was seen only when at least one of the partners was in a non-native state, which could be induced by a temporary pH change or immobilization of enolase in a phospholipid micelle. The non-native conformation of enolase bound also other proteins, such as albumin and enolases from other organisms [51]. Their study concluded that the moonlighting adhesiveness of enolase is not highly specific; which is in accordance with the multiple adhesion targets identified for bacterial enolases (Table 1). These results agree with the suggestion by Tompa and coworkers [2] that the structural flexibility of unstructured proteins or protein regions give rise to unprecedented molecular interactions that may lead to moonlighting. This hypothesis predicts that structural flexibility, not the structure *per se*, is critical for a moonlighting function in a protein.

It is possible that specific paralocous alleles of a gene encode moonlighting variants. Two enolase genes are present in the genome of *L. plantarum* [78] and both genes are expressed in standard growth conditions [49]. Enolase preparation from the surface of *L. plantarum*, apparently containing both enolases, bound plasminogen as well as fibronectin, [16] and mutagenesis studies showed that fibronectin-binding was performed by only one of the enolases [49]. The genome of *M. tuberculosis* contains two genes encoding the molecular chaperonins GroEL1 (also termed Cnp60.1) and GroEL2 (Cnp60.2), which both have impact on virulence of *M. tuberculosis* and induce host inflammatory responses [79]. Mutagenesis analyses revealed that GroEL2 is essential for survival of the bacteria whereas the *cnp60.1* gene could be inactivated; the mutated strain failed to produce granulomatous inflammation in either mice or guinea pigs [80]. GroEL1 of *M. tuberculosis* is released into culture medium during bacterial growth [81] and GroEL2 was identified among capsule-associated proteins on the bacterial surface [82]. Inhibition assays using recombinant GroEL2 protein as well as anti-GroEL2 polyclonal antibody showed that GroEL2 facilitates association of *M. tuberculosis* with murine macrophages [76], the receptor was identified as the CD43 molecule, a major sialoglycoprotein on the surface of human T lymphocytes, monocytes, granulocytes and some B lymphocytes [83]. Purified GroEL1 and GroEL2 proteins induce cytokine release *in vitro* from human peripheral blood mononucleated cells but show differences in their potency and mechanism [80,81,84]. It is generally considered that alveolar macrophage is the primary host niche of *M. tuberculosis* [81]; the observed interactions of the GroEL proteins with monocytes indicate their importance as virulence factors in the pathogenesis of tuberculosis. These results suggest that the two enolase variants and the two GroEL variants are moonlighting proteins but express differing functions. Overall, the possible presence of specific moonlighting protein-encoding alleles in bacteria remains open.

## 4. Are Moonlighting Proteins Secreted or Are They Released from Traumatized Cells?

The moonlighting proteins have been called “surface-associated housekeeping enzymes”, “anchorless surface proteins”, or “unconvential secreted proteins”, which all refer to the fact that their sequences do not contain known sequence motifs for secretion or anchoring onto the bacterial or the eukaryotic cell surface [1,10,85]. The mechanisms of how moonlighting proteins translocate to the cell exterior have remained open. Alternatives are that they are released from dead or traumatized bacterial cells and then bind onto the surface of neighboring cells, or that they are secreted onto the cell surface by an as yet-undescribed mechanism. In bacteria, experimental evidence for both hypotheses have been described, but overall, the data remains scattered and only suggestive, also, the two alternatives are not fully mutually exclusive.

The list of moonlighting adhesion proteins (Table 1) is comprehensive, and the proteins do not show any obvious, over-all conformational relatedness that would explain their translocation to cell surface. The secretion hypothesis has been put forward by the observations that genetic fusion of a C-terminal hydrophobic tail of 12 amino acids to GAPDH prevented its export to the surface of *S. pyogenes* [86]. In enolase of *Escherichia coli*, substitution of Lys341 at an automodification site (see below) with other amino acids abolished its export to cell surroundings [87], whereas in *Bacillus subtilis* enolase deletion of a central hydrophobic α-helical domain of 19 amino acids abolished surface translocation [88]. Further, an accessory *secA2* gene in *Listeria monocytogenesis* is involved in the secretion of enolase and other proteins [89], but a homologous gene is lacking in several bacterial species, such as lactobacilli that express moonlighting enolase and often have multiple gene copies. Enolases of *E. coli*, *Enterococcus faecalis*, and *B. subtilis* are partially automodified covalently by their substrate 2-phosphoglycerate, and in *E. coli*, an amino acid substitution at the modification site abolished translocation of enolase into the medium [87], which suggests that posttranslational modifications could have a role in translocation of moonlighting surface proteins. 

There is also evidence suggesting that moonlighting proteins are released by stressed cells in a bacterial population and reassociate at favorable conditions onto the surface of the same or neighboring cells or other surfaces. Spontaneous lysis of *B. subtilis* cells at stationary growth phase leads to leakage of 5% of the activity of isocitrate dehydrogenase, considered to be a cytoplasmic enzyme marker [90]. Chloramphenicol or a proton motive force inhibitor did not inhibit translocation of several moonlighting proteins of *B. subtilis* into the stationary-phase growth medium [88]. In *L. plantarum*, the amount of surface-associated GAPDH activity correlates with the growth phase and with increase in membrane permeability [91], and growth of *Bifidobacterium animalis* subsp. *lactis* with bile acids increased levels of DnaK on the cell surface [64]. DnaK is a molecular chaperone with multiple adhesive and immunostimulatory moonlighting functions (Table 1). Binding at low pH of extracellular moonlighting proteins onto the surface of *Lactobacillus crispatus* as well as their release from *L. crispatus* surface by alkaline stress [28,92] have been reported. GAPDH of group B streptococci (GBS) was not released into medium by pilus mutants that display a lower level of cell lysis; on the other hand, factors enhancing cell lysis, such as Triton X-100, penicillin, and overexpression of an autolysin, enhanced the amount of GAPDH at the cell surface as well as in the culture supernatant [29]. These results favor the hypothesis that moonlighting proteins detach from damaged cell walls or leach from lysed cells, however, they do not explain how the moonlighting proteins are translocated to the cell wall. 

To summarize, the translocation mechanism(s) of moonlighting proteins remain unknown, and we do not know whether one or more mechanisms are involved. The studies have been performed with different organisms and different proteins which complicates obtaining a mechanistic general view. 

## 5. Adhesive Properties in Bacterial Moonlighting Proteins

The adhesive functions of moonlighting proteins include adhesion to host epithelial cells, mucus and extracellular matrix (ECM) components, as well as interaction with circulating host components and modulation of host immune responses (Table 1). It is interesting that, when the target surfaces (epithelial cells, mucus) or a limited number of target molecules (ECM proteins, circulating proteins) are considered, several adhesive moonlighting proteins exhibit similar *in vitro* functions in pathogenic and commensal bacteria (Table 1). This supports studies on the concept of surface exclusion of pathogens [93,94,95] by commensal bacteria, where the highly numerous bacteria in the microbiota prevent pathogen entry by competing for colonization and space on mucosal surfaces or for physiologically important circulating proteins. This has mostly been addressed with epithelial cell lines *in vitro*, where, e.g., *L. plantarum*, *Lactobacillus fermentum* and *Lactobacillus jensenii* were found to use moonlighting proteins in competitive exclusion and displacement of pathogens [30,96,97]. Whether the surface exclusion is based on recognition of the same receptor molecules or whether it is based on a less-specific steric hindrance remains yet open. 

Another frequently studied adhesive function of bacterial moonlighting proteins is their ability to act as a plasminogen/plasmin receptor. Immobilization of plasminogen on bacterial surface, onto plasminogen receptors, enhances its proteolytic activation by human activators to plasmin, a potent serine protease, and also protects plasmin from inactivation by the circulating inhibitor α2-antiplasmin (α_2_AP) [98,99]. The process enhances surface-bound proteolytic activity which can advance bacterial survival or spread in the host [99]. Plasmin is broad spectrum protease which degrades fibrin, extracellular matrices, and activates human enzymes attacking connective tissue components [100]. The interaction with plasminogen may also increase bacterial adhesiveness. Plasminogen binds to bacterial cell surface receptors as well as to integrin molecules on eukaryotic cells and can thus also enhance bacterial adherence to host epithelia by a bridging mechanisms [101,102,103]. Plasminogen, but not plasmin, mediates *in vitro* adherence of *S. pneumoniae* to pulmonary epithelial and vascular endothelial cells. Transmigration of *S. pneumoniae* across endothelial and epithelial cell layers was potentiated by damage of intracellular junctions by active plasmin [104], which may promote the migration of pneumococci through tissue barriers. Plasminogen bound on surface of human respiratory epithelial cell lines and on brain-derived endothelial cells supported *in vitro* adherence of pneumococci but did not increase pneumococcal invasion into the target cells [105]. Plasminogen increased adherence as well as β-1integrin-dependent invasion of *S. pyogenes* to keratinocytes [102], and moonlighting plasminogen receptors are important virulence factors also for group A streptococci [86,106]. However, they also occur (Table 1) on a surprisingly high number of commensal bacteria species, where their biological functions remain largely unknown 

### 5.1. GAPDH

The two best characterized bacterial moonlighting enzymes are GAPDH and enolase (Table 1). GAPDH is involved in bacterial adhesion to several host components: blood group antigens, the cytoskeleton proteins actin and myosin, the serum proteins albumin, fibrinogen and plasminogen, the ECM proteins, as well as colonic mucins. Targets for GAPDH also include epithelial and endothelial cells, and it has been associated with several physiological and immunological functions (Table 1). GAPDH of *S. pyogenes* has been identified as an ADP-ribosylating enzyme [102]. ADP-ribosylation of host components is common for bacterial toxins [103]. Streptococcal cells as well as surface GAPDH activate the protein tyrosine kinase and the protein kinase C in human pharyngeal cells, which indicates that GAPDH regulates phosphorylation of cellular proteins and may play a role in pathogenesis and in cellular communication between streptococci and the host [107]. 

Bacteria may also use GAPDH to evade and modulate the host immune system. GAPDH of *S. pyogenes* binds C5a and inhibits C5a-activated chemotaxis and H_2_O_2_ production by human neutrophils [37], and GAPDH of *S. agalactiae* is a virulence-associated immunomodulatory protein [108] that induces B and T cell activation of spleen lymphocytes *in vitro*. The elevated IL-10 production induced by recombinant GAPDH seems to be important for successful colonization of the host by *Streptococcus agalactiae* [108]. Also the GAPDH of *S. pneumoniae* was reported to be antigenic in humans, and vaccination of mice with recombinant GAPDH elicited protective immune response against respiratory challenge with virulent pneumococci [109].

Genetic evidence also suggests that GAPDH is a virulence factor for *S. pyogenes*. Fusion of a hydrophobic tail into the C-terminus of GAPDH prevented the surface translocation and diminished plasminogen binding to the bacteria as well as bacterial adhesion to pharyngeal cells [86]. The mutant strain was completely attenuated for virulence in a mouse peritonitis model, and surface expression of GAPDH thus seems mandatory for the virulence of *S. pyogenes* [106].

### 5.2. Enolase

Enolase has been reported to bind mucus and epithelial cells, but its best-described moonlighting activity is its function as a plasminogen receptor (Table 1). Invasion of pneumococci through basement membranes is thought to be important in meningitis, and plasminogen activation by host activators was found to potentiate pneumococcal penetration through the reconstituted basement membrane preparation Matrigel [110]. Soluble recombinant enolase binds *in vitro* at neutral pH to cell surface of *S. pneumoniae* when associated with plasminogen [39], and pneumococcal enolase-coated microspheres degraded radiolabeled ECM and Matrigel [111]. Also enolase of *Bacillus anthracis* binds plasminogen and bacteria incubated with plasminogen together with streptokinase (a plasminogen activator) were *in vitro* capable of degrading fibronectin [38]. Pneumococci expressing enolase substituted for the internal plasminogen binding site show reduced virulence in a mouse model of intranasal infection [74], and immunization of mice with enolase of *S. suis* protects against streptococcal infection [53].

It was recently found that pneumococci use enolase also to evade the host immune system [54]. Pneumococcal enolase binds C4b-binding protein (C4BP) and thereby protects bacteria from complement-mediated killing. Interestingly, plasminogen and C4BP bind to different domains of the enolase protein indicating that enolase has a dual role in pneumococcal pathogenesis. Immunization of rats with enolase from the oral pathogen *Streptococcus sobrinus* increased the levels of salivary IgA and IgG antibodies, and recombinant enolase was considered a promising candidate as a vaccine against dental caries in humans [112].

### 5.3. Molecular Chaperones

Molecular chaperones are a major class of bacterial moonlighting proteins. Their canonical function is to bind and assist folding or unfolding of macromolecular structures, and, given the prevailing nature of moonlighting in bacteria, it is not surprising that a variety of moonlighting functions have been identified for molecular chaperones. The bacterial Hsp70 protein, DnaK, was identified in the cell wall proteome of *L. monocytogens* [21]. Moonlighting functions of DnaK have been intensively studied in *Mycobacterium tuberculosis.* Recombinant DnaK binds human plasminogen [59], stimulates monocyte chemokine synthesis by binding to the CD40 receptor [113] and dendritic cell maturation by binding to chemokine receptor CCR5 [65]. CCR5 is also used by HIV as a co-receptor, and by binding the same receptor DnaK can compete with HIV for the binding [65,66]. Virus infection of activated CD4^+^ T cells was inhibited by the C-terminal peptide 407–426, which can have potential as a novel strategy to block the binding of HIV to its receptor [66]. As in *M. tuberculosis*, GroEL of *L. johnsonii* has an immunomodulatory activity and stimulates interleukin-8 secretion in human macrophages *in vitro*. It also aggregates cells of the human-specific gastric pathogen *Helicobacter pylori* [68] and thus can enhance clearance of the pathogen from gastric mucosa.

## 6. Moonlighting Proteins of *Lactobacilli*: Ionic Interactions Are Important in Anchorage and Activity

Species of *Lactobacillus* are fermentative and secrete lactic acid as a primary metabolite and thereby rapidly acidify their environment down to pH 4. These bacteria commonly inhabit acidic environments, such as human small intestine and vagina [114]. Several moonlighting proteins have been identified in species of *Lactobacillus*, these include GAPDH, enolase, glucose-6-phosphate isomerase (GPI), triose phosphate isomerase, phosphoglycerate kinase, elongation factor Tu, glutamine synthetase (GS) as well as GroEL [17,27,28,30,32,34,49,68,115]. These proteins exhibit a wealth of adhesive properties (Table 1) whose biological relevance, however, remain largely unidefined.

**Figure 1:**
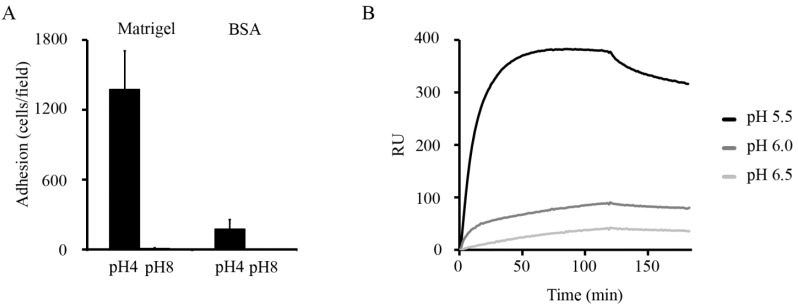
Adherence of *L. crispatus* ST1 and binding of His_6_-GS of ST1 are affected by pH. (**A**) Adherence of *L. crispatus* ST1 at pH 4 and pH 8 to the immobilized basement membrane preparation Matrigel and to BSA. Prior to the assays, the bacteria from an overnight culture were washed with the pH 4 or the pH 8 buffer, and the adhesion assays were performed at the corresponding pH. Means of adherent bacteria in 20 randomly chosen microscopic fields of 1.6 × 10^4^ μm^2^ and standard deviations are shown. (**B**) Binding of purified His_6_-GS to immobilized laminin was tested by surface plasmon resonance at pH 5.5, 6.0 and 6.5 (Adapted from [28] Copyright^©^ American Society for Microbiology).

**Figure 2:**
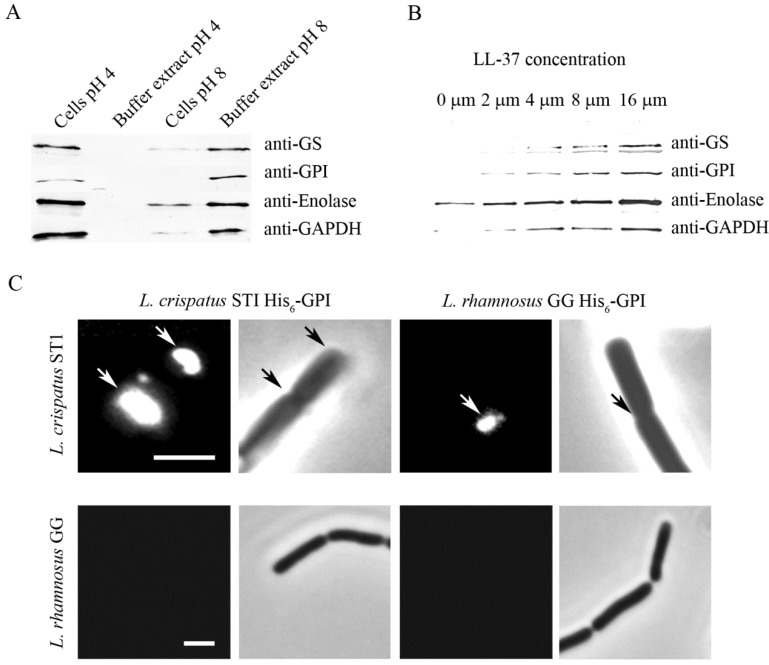
Release of moonlighting proteins from *L. crispatus* ST1 surface and reassociation of recombinant proteins onto the cell wall. (**A**) Western blotting of GS, GPI, enolase, and GAPDH from the surface of *L. crispatus* ST1 cells and from the cell-free buffer. The cells were incubated at pH 4 or pH 8 for 1 h, and the moonlighting proteins in neutralized samples were visualized by immunoblotting. (**B**) Release of moonlighting proteins in the presence of increasing concentrations of the cationic peptide LL-37. *L. crispatus* ST1 cells were treated with LL-37 at the indicated concentrations, and the moonlighting proteins in the buffer were visualized by Western blotting. (**C**) Binding of His_6_-GPI proteins of *L. crispatus* ST1 and *L. rhamnosus* GG to bacterial cells at pH 4. The binding was visualized by indirect immunofluorescence, phase contrast images of the microscopic field are also shown. The arrows indicate protein binding to bacterial cell surface. Size bar, 3 µm. (Adapted from [28] Copyright^©^ American Society for Microbiology).

Recent *in vitro* evidence indicates that ionic interactions are critical in surface anchorance and in adherence activity of moonlighting proteins of lactobacilli. *L. crispatus* inhabits human vagina where it is thought to exert beneficial, probiotic effects and diminish inflammatory vaginosis, possibly through adhesion-associated mechanism [116]. The strain ST1 of *L. crispatus* shows pH-dependent adhesion to Matrigel *in vitro* (Figure 1A) [28] and in binding to human plasminogen [92]. The low adhesiveness at neutral or basic pH seems to depend on two aspects, (i) the anchoring of the adhesins on the cell surface, and (ii) their binding activity. First, several adhesive moonlighting proteins, *i.e.*, enolase, GAPDH, GS, and GPI of *L. crispatus* ST1 are detached from cell surface at neutral or basic pH but remain surface-bound at pH 4 (Figure 2A) [17,28,92]. This seems to also hold for several other species of *Lactobacillus* [17,47]. These proteins have isoelectric points around 5 and thus a positive net charge at pH values below 5, a condition where purified enolase and GAPDH of *L. crispatus* ST1 bind *in vitro* to negatively charged lipoteichoic acids as well as to the *L. crispatus* ST1 cell surface [28,92]. Lipoteichoid acids are common surface constituents in Gram-positive bacteria, and the results suggest that they may anchor moonlighting proteins on the bacterial surface via ionic interactions. The role of ionic interactions in the anchoring is further indicated by the findings that moonlighting proteins are released from the *L. crispatus* ST1 surface in 0.25 mM sodium or choline chloride [92] and that the release is increased by μM concentrations of LL-37 (Figure 2B) [28]. LL-37 is a cationic antimicrobial peptide that is part of innate immune defense against bacteria and produced mainly by epithelial cells and phagocytes [117]. Contrary to moonlighting proteins of *L. crispatus* ST1, the abundant S-layer proteins of *L. crispatus* and other lactobacilli have a pI of *ca.* 10 [118], and they are not released from cell surface at neutral or basic pH [92].

At pH 4, but not at pH 8, purified His_6_-tagged GS and GPI showed a localized *in vitro* binding onto *L. crispatus* ST1 cells and bound to cell division sites and cell poles (Figure 2C) [28]. The cell division septa have structural and functional specifics [119,120,121] and it is tempting to speculate that moonlighting proteins are translocated to cell surface via these structures, either by diffusion or by a transport system. It is interesting that moonlighting proteins of *L. crispatus* ST1 and of the well-studied probiotic *Lactobacillus rhamnosus* GG bound *in vitro* to *L. crispatus* ST1 cells but not to *L. rhamnosus* GG cells (Figure 2C) [28]. The basis of this difference remains to be defined but the observation, however, suggests that differences in cell surface architecture and chemical composition influence the amount of moonlighting proteins bound on bacterial cell surfaces.

Another reason for poor adhesiveness at pH 8 by *L. crispatus* ST1 cells (Figure 1A) is that the moonlighting proteins of *L. crispatus* ST1 bind more efficiently at low pH [28]. Matrigel is composed of mainly laminin and type IV collagen [122], and it was found that purified His_6_-GS of *L. crispatus* ST1 bound to laminin much better at pH 5.5 than at pH 6.5 (Figure 1B) [28]. Further, His_6_-tagged GS and GPI showed a similar pH-dependency in binding to type I collagen, which is the most abundant protein of mammalian ECMs, but failed to bind to the ECM proteins type IV collagen and fibronectin. Increased binding at acidic pH has been also observed for mucus-binding proteins of *Lactobacillus reuteri* [123] and in the binding of enolase and GAPDH of *L. plantarum* [16] as well as of EF-Tu [70] and GroEL [68] of *L. johnsonii* to epithelial cells and mucins.

Saad and coworkers [91] reported that, at acidic pH, the amount of surface-associated GAPDH on *L. plantarum* cells correlates with stationary growth phase and increased cell permeability. Similarly, stress by alkaline pH or by LL-37 increases cell wall permeability and release of moonlighting proteins from the surface of *L. crispatus* ST1 cells (Figure 2B) [28]. LL-37 is a cationic antimicrobial peptide produced by epithelial cells and phagocytes and disrupts bacterial cell wall integrity by binding to lipid molecules [124]; whether it merely facilitates release of moonlighting proteins from traumatized cells or whether it also potentiates their transfer to cell surface, remains to be studied. Stress induced by growth of bifidobacteria in the presence of bile acids [64] and of streptococci under iron starvation [125] increases the amount of moonlighting proteins in the cell exterior. These findings indicate that stress is a determinant of the translocation of moonlighting proteins in several Gram-positive bacteria.

## 7. Adhesive Moonlighting Goes in Several Directions: Multitasking in Fimbriae, Flagella, and Surface Proteases

Fimbrial filaments are composed of hundreds of copies of the major fimbrillin subunit and a few copies, often located at tip of the filament, of minor proteins that are responsible for the adhesive, lectin-like characteristics of the fimbrial types [126,127]. Several fimbrial types of *E. coli* and *Salmonella enterica* immobilize plasminogen on the bacterial surface in a lysine-sensitive manner and enhance its activation, thus localizing the bacterium-plasmin complex on target tissues recognized by the bacterial lectin [128,129,130]. The molecular details of these interactions have remained mechanistically uncharacterized; it is however well established that fimbrial interaction with plasminogen is not based on lectin-like properties and thus can be considered moonlighting by the filamentous structure. In the S-fimbria associated with *E. coli* strains causing newborn meningitis, genetic studies indicated that the interaction involves a fimbrial minor subunit other than the sialic acid-binding lectin subunit [130]. Curli is a common filamentous adhesin expressed by *E. coli* and *S. enterica* and shown to interact with human contact phase proteins [131], which are involved in fever, hypotension and bleeding disorders associated with the infections. These examples show that fimbrial filaments affect bacterial colonization and pathogenicity also via functions other than their adhesiveness to host tissues. Also, flagellar filaments immobilize plasminogen apparently via lysine residues exposed on the filament surface and thus increase proteolytic activity on the cells [132]. Flagella are associated with bacterial mobility but also possess adhesive properties. Flagella of *Pseudomonas aeruginosa* bind to heparan sulphate proteoglycans at the basolateral surface of airway epithelium [133] and to human Le^x^ and sialyl Le^x^ determinants in human respiratory mucins [134]. Flagella of *S. enterica* moonlight not only in plasminogen binding [129] but also bind to and enhance biofilm formation on cholesterol-containing surfaces [135]. The latter was concluded to enhance the chronic carrier state of the bacteria on gallstones. Flagella of enteropathogenic (EPEC), enterohemorrhagic (EHEC), meningitic, enterotoxigenic, and probiotic *E. coli* strains have been identified to mediate bacterial adhesion to epithelial cells [136,137,138,139,140]. These flagella are of different serotypes, and the identified molecular targets include human or animal gut mucins. Interestingly, the EPEC and the EHEC flagella differ in binding to collagen, laminin and fibronectin [137]. Fimbriae and flagella are multimeric protein complexes, and the subunits active in the above activities have not yet been identified, hence it is open whether they represent true moonlighting of a single protein species.

The classical example of a surface protease with moonlighting adhesive functions is the Hap hemagglutinin/protease secreted by *Vibrio cholerae* [141], which perturbs pericellular barrier function in epithelial cells by degrading tight junctions [142] and by hydrolyzing fibronectin and ovomucin on human epithelial cells [143]. Hap is a metalloproteinase that also degrades lactoferrin and nicks enterotoxin as well as agglutinates chicken erythrocytes and participates in bacterial attachment to gut epithelium. These properties are thought to enhance spread of the cholera bacterium within the human intestine. Pla is a surface protease—a plasminogen activator—and a dramatic virulence factor of the plague bacterium *Yersinia pestis* that destroys control of the hemostasis balance in humans [144]. Pla increases virulence of *Y. pestis* also by mechanisms that do not involve proteolysis. It mediates adhesion to laminin of basement membranes [145], which is sensitive to plasmin activity created by e.g., Pla itself. Degradation of laminin is thought to loosen tissue barriers and enhance migration of bacteria at early stages of plague. Another non-proteolytic mechanism of how Pla enhances bacterial dissemination is invasiveness to alveolar macrophages, where Pla utilizes the C-type lectin receptor DEC-205 for invasion [146]. 

These findings demonstrate that moonlighting, or multifunctionality, in bacteria goes in several directions, metabolic enzymes moonlight as adhesins and as immunomodulators, canonical adhesion proteins moonlight with host circulating effector proteins, and taxis proteins and proteases moonlight as adhesins and/or invasins.

## 8. Conclusions

It has become evident that moonlighting, or the capacity to perform biological functions not related to the canonical function assigned to the protein, is common in bacterial proteins. Moonlighting was originally considered a virulence trait in pathogenic bacteria, but the same protein species with moonlighting functions have been described in pathogens and in members of the human or the animal microbiota. A general conclusion we wish to stress is that moonlighting proteins appear to be part of bacterial routine in communication with the environment and in responses to environmental changes or stress. The findings that moonlighting proteins released from a single bacterial species can reassociate onto the surface of another species [28,29,68] can be seen as a novel mechanism in bacteria-bacteria interactions and may have importance in bacteria-host interactions.

**Figure 3:**
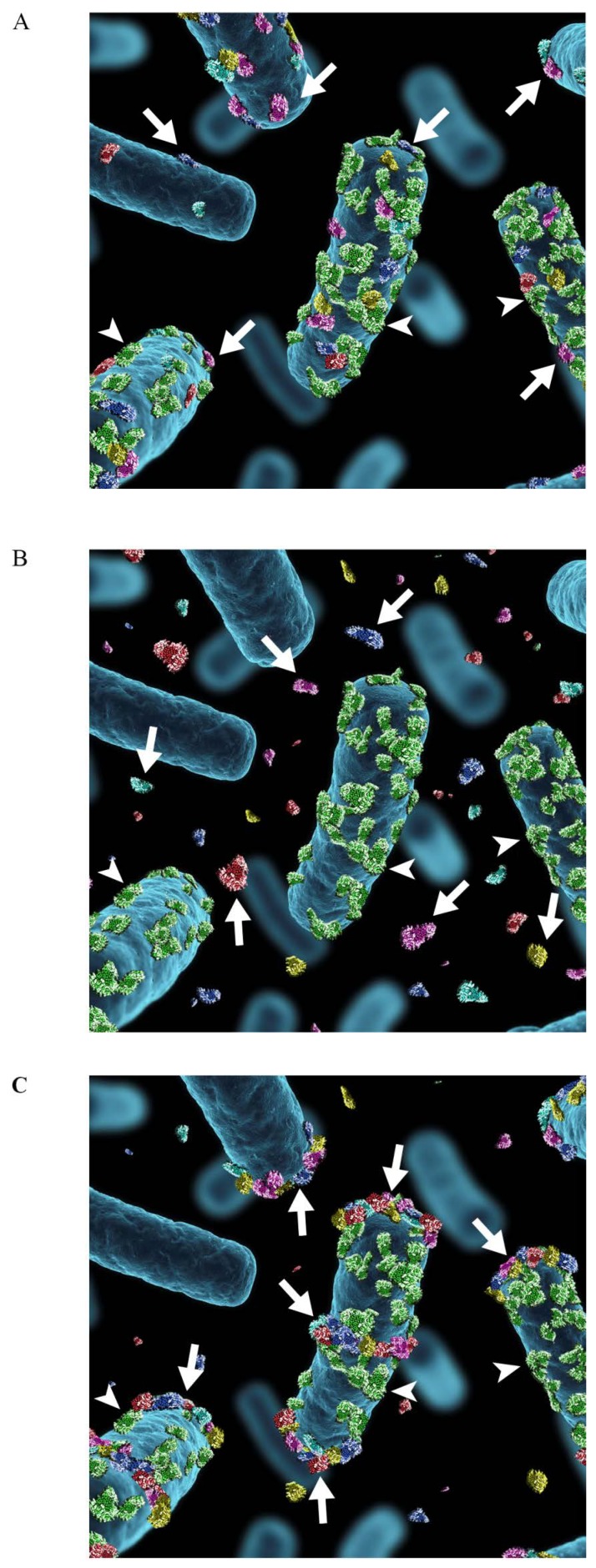
Schematic illustration of the association of moonlighting proteins with the cell surface of *Lactobacillus.* (**A**) Moonlighting proteins (shown in red, blue, yellow, purple and turquoise) of *Lactobacillus* associate to the cell surface via electrostatic or ionic interactions (**B**) and they are released into surroundings in stress situations, such as neutral or alkaline pH or presence of cathelidicins or bile acids. The surface location of the S-layer protein (shown in green) with the pI of 10, is not affected by environmental changes. (**C**) The extracellularly released moonlighting proteins associate back onto the cell surface in favorable environmental conditions, *i.e.*, at acidic pH. The proteins associate with the cell surface of the same lactobacillar species where the proteins were originally released, but also with the cell surface of other lactobacillar species. The binding is not evenly distributed around the cells but is concentrated to cell division areas as well as to the cell poles. The arrows indicate the moonlighting proteins and arrowheads the S-layer protein.

Our hypothesis is, that the moonlighting proteins in pathogenic species and in commensal lactobacilli are functionally different because they are anchored differently on the bacterial surface. Several pathogenic species seem to retain moonlighting proteins on cell surface at neutral pH [38,39,41,42,45,50,53,60], whereas this is not the case for several lactobacilli [16,28,32,47,92]. The latter is illustrated for *L. crispatus* in Figure 3. Moonlighting proteins remain cell-bound by ionic interactions and are highly adhesive at low pH, whereas at neutral or basic pH they are detached from the cell surface into the medium, where they may inhibit bacterial adhesion or have other interactions with human cells or tissues. Also, in the case of *L. crispatus* ST1 described here, the bacterium has other adhesins that are surface-bound by the sortase mechanism [147] and function well at neutral pH [148,149]. The detachment of moonlighting proteins from *L. crispatus* cells is enhanced by low concentrations of epithelium-derived cationic peptides such as LL-37, which suggests that the anchoring/detachment phenomenon may take place *in vivo*. Binding and activation of the human plasminogen is an established virulence function in several bacterial infections [99]. *Lactobacillus* is regarded as a safe organism for humans and a member of the microbiota, but cells of several *Lactobacillus* species very efficiently enhance activation of plasminogen at neutral pH. However, unlike with several pathogens (reviewed in Bergmann and Hammerschimdt, 2007 [150]; Lähteenmäki and coworkers [99]), the activity at neutral pH is in the cell-free buffer and not protected from α_2_-antiplasmin [17,28,47]. This means that the plasmin created in the presence of lactobacillar cells remains proteolytically active only when complexed onto a near-by lysine-containing surface, such as the fibrin clot which is the main physiological target for plasmin proteolysis [151,152]. Indeed, a lowered fibrin deposition in the presence of lactobacilli has been observed in mice suffering from pneumococcal lung infection [153,154]. These findings exemplify a mechanism of how bacterial species may differ in the biology of a moonlighting function and stress the importance of our understanding of the mechanisms and the efficiency of surface anchoring of moonlighting proteins.
